# MALDI-TOF MS Profiling-Advances in Species Identification of Pests, Parasites, and Vectors

**DOI:** 10.3389/fcimb.2017.00184

**Published:** 2017-05-15

**Authors:** Jayaseelan Murugaiyan, Uwe Roesler

**Affiliations:** Institute of Animal Hygiene and Environmental Health, Centre for Infectious Medicine, Freie Universität BerlinBerlin, Germany

**Keywords:** MALDI MS typing, MALDI TOF MS, intact protein profiling, pests, parasites, species, spectra reference databases

## Abstract

Invertebrate pests and parasites of humans, animals, and plants continue to cause serious diseases and remain as a high treat to agricultural productivity and storage. The rapid and accurate species identification of the pests and parasites are needed for understanding epidemiology, monitoring outbreaks, and designing control measures. Matrix-assisted laser desorption ionization time-of-flight mass spectrometry (MALDI-TOF MS) profiling has emerged as a rapid, cost effective, and high throughput technique of microbial species identification in modern diagnostic laboratories. The development of soft ionization techniques and the release of commercial pattern matching software platforms has resulted in the exponential growth of applications in higher organisms including parasitology. The present review discusses the proof-of-principle experiments and various methods of MALDI MS profiling in rapid species identification of both laboratory and field isolates of pests, parasites and vectors.

## Introduction

Invertebrate pests and parasites of plants, mammals, birds, amphibians, and reptiles account for increased losses in the agricultural sector and continue to play a considerable role in the spread of infectious diseases (Paini et al., 2016; Poulin et al., 2016). Accurate and rapid species identification of pests and parasites is extremely important for initiating species-specific treatment procedures, understanding the epidemiology, monitoring of outbreaks, and designing control measures (Gibson, 2009; Furlong, 2015). Traditionally, trained taxonomists or entomologists visually examine or observe microscopically the morphological characteristics for species determination. However, in addition to being time-consuming, misidentification possibilities, distinguishing immature or development stages, damaged samples, cryptic species, and species differing by minor morphological characteristics make identification challenging and often impossible (McKeand, 1998). Molecular methods, whichever available, are accurate and applicable to any development stages. On the other hand, these methods are also labor intensive, expensive, time consuming, and difficult to apply for species for which sequences are not available (Wong et al., 2014). Furthermore, in certain cases such as *Leishmania* subtyping, which is crucial for treatment, identification generally requires several weeks for performing complex and expensive analyses (Roelfsema et al., 2011). In recent years, proteome based linear matrix-assisted laser desorption/ionization time-of-flight mass spectrometry (MALDI-TOF MS profiling or MALDI MS typing), which is a well-established technique for microbial species identification, has also been successfully applied to a variety of parasites and their vectors (Seng et al., 2009; Bizzini and Greub, 2010; Patel, 2013; Nomura, 2015; Karger, 2016; Singhal et al., 2016; Yssouf et al., 2016). The popularity of the method is due to its rapidness, easy to use, high throughput analysis, accuracy, reliability equal to that of the molecular methods of species identification and cost-effectiveness despite the initial cost of the machine (Dhiman et al., 2011; Neville et al., 2011; Tran et al., 2015; Ge et al., 2016). The technique involves generation of MALDI MS spectra for a given species and pattern matching with that of the spectra database of the well-defined species to deduce the species information (Welker and Moore, 2011; Nagy et al., 2012; Suarez et al., 2013). The spectra pattern matching is carried out using automated commercial software suites, such as Bruker Biotyper (Bruker Daltonics, Bremen, Germany), VITEK MS (BioMérieux, Nuertingen, Germany: earlier Axima (Shimadzu)-SARAMIS (AnagnosTec) systems), Andromas (Andromas SAS), or MicrobeLynx (Waters) (Sogawa et al., 2011; Bille et al., 2012; Patel, 2013; Cassagne et al., 2016). The commercial software tools are usually integrated with their own spectra reference database, and utilize a unique algorithm for spectra processing, pattern matching, and result interpretation.

The manufacturer-provided database is limited only in terms of reference spectra for available microbial species, and currently the reference information for pests and parasites are not included. The software generally includes the possibility to create reference spectra for any organism to be integrated within the existing database. The database extension has been utilized to create additional reference spectra to enhance the identification confidence and to include reference spectra of the missing species, including higher organisms (Bohme et al., 2012; Murugaiyan et al., 2012, 2014; Hoppenheit et al., 2013). Several reviews have been dedicated to the recent developments of MALDI MS typing of plant nematodes and organisms related to parasitology (Ahmad and Babalola, 2014; Karger, 2016; Singhal et al., 2016; Yssouf et al., 2016). Therefore, the focus of this review is on the various approaches reported for MALDI MS typing based species identification of pests, parasites, and their vectors ranging from laboratory isolates to that of field samples.

## MALDI MS basis of species identification

MALDI MS based species identification involves three steps:

1. Sample spotting onto a specially designed metal plate called the target plate,
2. MALDI TOF MS measurments and
3. Species deduction through pattern matching of the spectra with the database of spectra derived from known and/or well-defined species.

A small portion of biological substances e.g., microbial colonies or a drop of intact/crude protein extracted using simple procedures is directly added to the target plate and allowed to air dry. Then, the sample spot is overlaid with a drop of an excess concentration of UV-absorbing small organic compounds, referred to as a matrix. There are several choices of matrix such as α-cyano-4-hydroxycinnamic acid (HCCA/CHCA), sinapinic acid (3, 5- dimethoxy-4-hydroxycinnamic acid) (SA), and 2,5-dihydroxybenzoic acid (DHB). HCCA is most frequently reported matrix; however, there is no universally recommended matrix.

In the MALDI instrument, a small region of the crystalline sample-matrix spot (usually 0.05–0.2 mm in diameter) is irradiated using a pulsed beam of a laser, generally a nitrogen beam with a wavelength of 337 nm is used in most commercial machines. The matrix absorbs the laser energy, and rapidly heats up resulting in desorption (vaporization) or structural decomposition of the proteins and protonation to form a hot dense plume of ablated gases and ions (Clark et al., 2013). Using an electric field, the ions are accelerated into a vacuum tube that terminates in an ion detector. The ions are usually of single charge and the acceleration voltage results in the same kinetic energy applied to every single charged ions, which results in separation of ions based on mass/charge (m/z) ration in the drift or vacuum tube.

The time of flight “TOF” of ions is recorded as MALDI spectra where the x-axis represents *m/z* ratio and the y-axis represents the intensity (or number) of same/similar ions. MALDI MS ions are singly charged, representing the non-fragmented parent ion mass, the resulting spectra is simple, and therefore, the species of unknown samples can be easily deduced after data processing and direct pattern matching with that of the spectra established from well-defined species compiled as a database. The identity of spectral peaks or protein sequence information is not important, as the species deduced by matching the protein profiles usually at a range of 2–20 kDa (2,000–20,000 m/z) (Evason et al., 2001; Sauer and Kliem, 2010; Welker and Moore, 2011; Karlsson et al., 2015; Cassagne et al., 2016).

## Advances in invertebrate pests, parasite, and vector profiling

**Figure 1:**
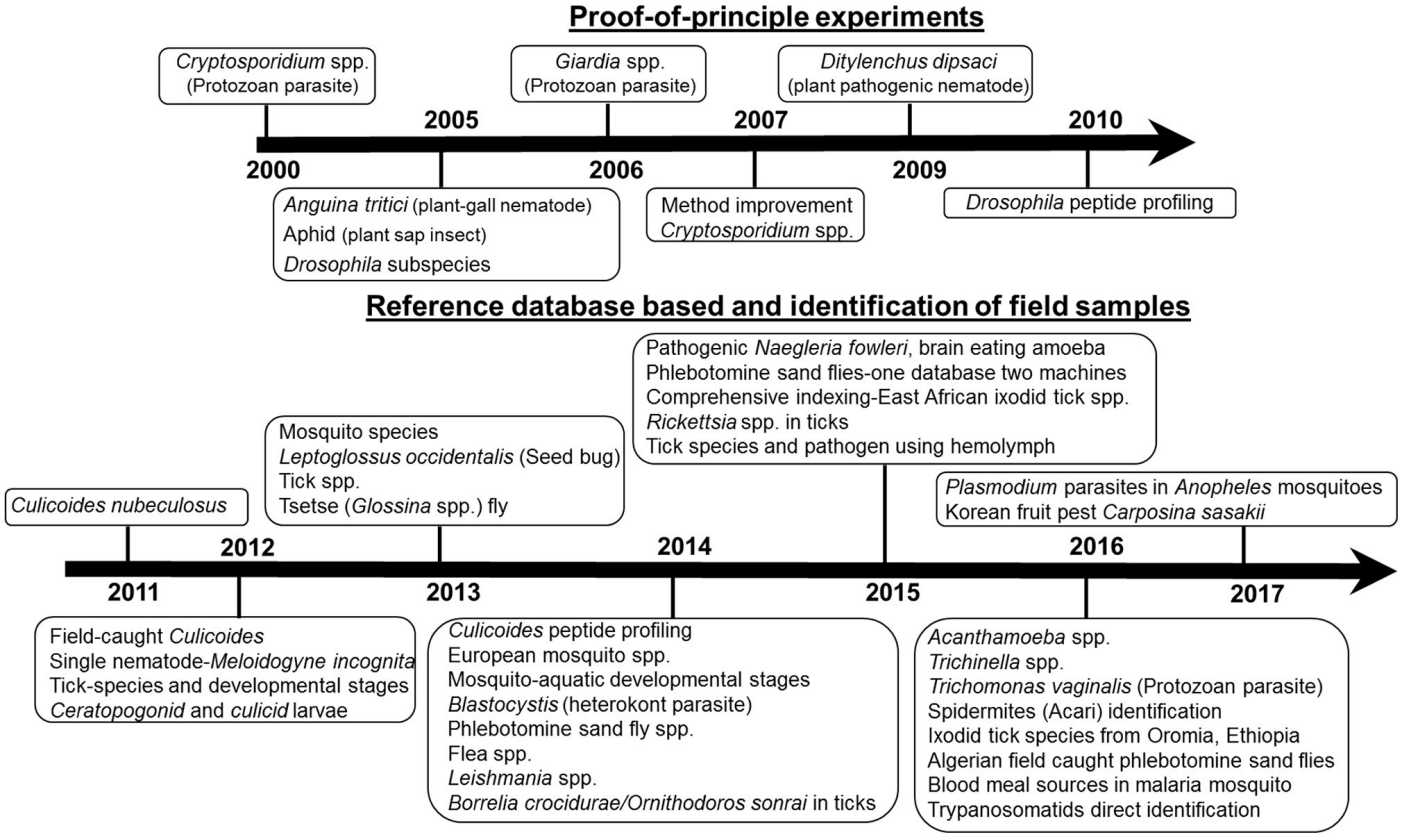
**Time line on MALDI profiling application of pests, parasites, and vector species identification**.

In the past 16 years (Figure 1), MALDI MS profiling has been successfully applied for species identification of different pests, parasites and vectors such as nematodes, protozoa, and arthropods.

### Proof of principle experiments

The early proof of principle experiments were focused on identification of biomarker peaks, standardization of sample preparation, matrixes and measurement optimization.

#### Protozoans and unicellular parasites

The first report of MALDI MS typing of protozoa was demonstrated using *Cryptosporidium* spp. associated with human infections. The species-specific spectra were reported from oocytes of *C. parvum* and *C. muris* isolated from feces of experimentally inoculated mice, lysed by freeze-thaw cycle, and spotted with HCCA as matrix (Magnuson et al., 2000). Later, it was shown that incubation of intact oocytes and purified sporozoites for 45 min with the matrix was critical for generating mass spectra with a large number of reproducible peaks for *C. parvum* oocysts (Glassmeyer et al., 2007). Subsequently, direct application of whole spores, spore shells, and soluble fractions of spore-forming unicellular parasites such as microsporidia, *Encephalitozoon cuniculi, Encephalitozoon hellem, Encephalitozoon intestinalis*, and *Brachiola algerae* isolated from humans and propagated on monolayers of Vero monkey kidney (E6) cells, displayed species-specific markers in the mass range of 2,000–8,000 Da (Moura et al., 2003). Later, species-specific peaks in the range of *m/z* 3,000–19,000 was reported for the water-borne protozoan parasite, *Giardia* spp., the causative agent of giardiasis. The cysts, cyst walls, and trophozoites of *G. lamblia* and *G. muris* isolated from feces of experimentally challenged mice, were washed, mixed with an equal volume of sinnapinic acid, incubated, and spotted for MALDI MS analysis (Villegas et al., 2006).

#### Insects and pests

Although not a pest or parasite, *Drosophila* has been used as a model for insect profiling possibilities. Protein extraction by simple grinding of adult whole insect in water was shown to generate species-specific spectra capable of distinguishing sibling species of *Drosophila* sub-species. The insect sex and matrix was not found to influence the spectra (Campbell, 2005). Likewise, species-specific peaks of varying intensities in a range of 3,000–25,000 *m/z* have been reported for three different aphids (plant phloem sap-feeding insects), green peach aphid *Myzus persicae* Sulzer, cowpea aphid *Aphis craccivora* Koch, and blue-green aphid (blue alfalfa aphid) *Acyrthosiphon kondoi* Shinji, independent of their dietary host plants (Perera et al., 2005).

#### Nematodes and developmental stage discrimination

Species-specific and diagnostic peaks have also been reported for simple extracts of three plant nematodes, *Anguina tritici* (wheat seed-gall nematode), its closely *Anguina funesta* (*r*yegrass nematode), and *Meloidogyne javanica* (root-knot nematode that infects horticultural and vegetable crops; Perera et al., 2005). Several years later, direct crushing of the root-knot nematode, *Meloidogyne incognita* was shown to be useful in rapid discrimination between the harmless and harmful J_2_ developmental stages and adult nematode (Ahmad et al., 2012).

### Peptide profiling

In this approach, also referred as shotgun mass mapping or SMM, whole body protein extracts were subjected to trypsin digestion without purification or fractionation steps, and the resulting peptides were utilized for generation of MALDI MS spectra for insect vector species such as *Drosophila* (Feltens et al., 2010) and biting midges (Uhlmann et al., 2014). Feltens et al. had applied nano-high performance liquid chromatography coupled with electrospray ionization mass spectrometry for identification of some of the MALDI MS profiles and revealed that most of the proteins were of muscles and mitochondria. However, SMM is time-consuming and handling large set of samples is challenging.

### Data independent analysis and clinical sample survey

The data independent species discrimination or grouping of microorganism is based on the visual examination for the presence or absence of peaks. This technique is very similar to those analyses performed before the days of the implementation of software with automated pattern matching algorithms; however, a different algorithm is used for rapid determination of the presence or absence of peaks for identification or discrimination. For example, this technique has been reported for the discrimination of the *Leishmania* subgenus *Viannia* or *Leishmania* exclusively based on the presence of 2 pairs of peaks (Mouri et al., 2014), as well as differentiation of protozoan parasitic *Entamoeba histolytica* and *Entamoeba dispar* (Azian et al., 2006).

### Database-based enabled rapid species identification extended to field samples

MALDI MS typing based rapid species identification is usually achieved through pattern-matching of the unknown samples with that of a spectral reference library (database) created from known organisms. The main concerns at the proteome level are differences between the various developmental stages and complexity associated with various body parts. As listed in Table 1, vector related reference spectra were reported using commercial MALDI instrument-software suites. The proteins were extracted through homogenization and the parameters recommended for microbial species identification was followed. HCCA and SA were reported as the most utilized matrices for Bruker Biotyper and SARAMIS (Vitex MS), respectively. Despite the success of the procedures, these reference spectra remain *in-house* databases. Following the compilation of vector-specific reference spectra databases, the method proved to be rapid (~2–5 min/sample) as in the case of microbial species identification.

**Table 1:** **Reference database based species identification of parasite vectors**.

**S. No**	**Organisms**	**Sample/database**	**Protein extraction ^†^**	Matrix^*^	**Software and database ^≠^**		**Comment**	**References**
					**Bruker BioTyper^±^**	**SARAMIS^¥^**		
1	*Culicoides* biting midges	Whole insects or thoraxes	(1) Water and (2) FA (5-50%)	SA, DHB		+	Two laboratory reared *-C. nubeculosus*, better spectra with SA, recommended removal of abdomen, ethanol storage, and high concentration of formic acid influenced spectra	Kaufmann et al., 2011
2		Thoraxes with head, wings, and legs	10% FA	SA		+	first field study, better results with fresh samples compared to ethanol stored	Kaufmann et al., 2012
3						+	Ethanol preserved larvae, Spectra differed with development stage	Steinmann et al., 2013
4				HCCA		+	field survey at Senegal, Africa	Sambou et al., 2015
5	Mosquitoes	head and thorax		SA		+	Several months of ethanol preserved	Muller et al., 2013
6		legs	70% FA and 50% ACN	HCCA	+		Proteins extracted from legs is sufficient	Yssouf et al., 2013b
7					+		European mosquito species identification	Yssouf et al., 2014a
8					+		Mosquitos' species and midgut microbial species identification after culturing	Tandina et al., 2016
9		Whole specimen	70% FA		+		Culicidae juvenile database-aquatic developmental stages	Dieme et al., 2014
10		Eggs		SA		+	Eggs as source for species surveillance	Schaffner et al., 2014
11		Abdomen	70% FA and 50% AC	HCCA	+		Blood mean source identification in malaria mosquito	Niare et al., 2016
12		Head and thorax	Water		+		Plasmodium parasite identification in *Anopheles*	Laroche et al., 2017
13	Ticks	Whole tick, nymphs, and larvae	Sonication with 6-M guanidinium chloride solution, acidified with TFA and eluted with 0.1% TFA/75% ACN	HCCA	+		Developmental stages for 7 tick species	Karger et al., 2012
14		Legs	70% FA and 50% ACN		+		Proteins extracted from legs is sufficient	Yssouf et al., 2013a
16			25% FA	SA		+	comprehensive indexing of East African ixodid tick species	Rothen et al., 2016
17		1 Spirochetes spotted directly 2 tick legs	70% FA and 50% ACN	HCCA	+		*Borrelia crocidurae* /*Ornithodoros sonrai* in ticks.	Fotso et al., 2014
18		1 Hemolymph from leg 2 *R. c. conorii* strain extracte	70% FA and 100% ACN		+		*Rh. sanguineus* specimens infected by *R. c. conorii* and *A. variegatum* specimens infected by *R. africae*	Yssouf et al., 2015a
19		1 Tick legs 2 Cultured and purified Rickettsia species			+		*Rickettsia* spp in Ticks	Yssouf et al., 2015b
20	Flea	Abdomen excised whole specimen	70% FA and 50% ACN		+		Five flea species	Yssouf et al., 2014b
21	*Glossina*. spp Tsetse fly	Whole insect and dissected body parts	70% FA and 100% ACN		+		Five lab reared *Glossina*. spp. Wings is sufficient	Hoppenheit et al., 2013
22	Phlebotomine sand flies	Thoraxes	(1) water and (2) 25% FA	SA	+		Discrimination of five Mediterranean species, Water extract yielded superior result to that of formic acid extract	Dvorak et al., 2014
23		Thoraxes, wings, and legs	70% FA and 50% ACN	HCCA	+		Six species caught from Algeria.	Lafri et al., 2016
24		Thoraxes with wings and legs	10% FA	SA, DHB		+	1. Ultraflex III MALDI TOF mass spectrometer measurement and an in-house phyton script to transfer mzXML files in to SARAMIS software. 2. Spectra measurement using Axima Confidence to create a reference spectra database for 20 species	Mathis et al., 2015

† Protein extraction by homogenization: FA, Formic acid; ACN, acetonitrile; TFA, Trifluroacetic acid;

* Matrix: HCCA, α-Cyano-4-hydroxycinnamic acid; SA, Sinapinic acid; DHB, 2,5-Dihydroxybenzoic acid; Software and database:

≠ *Plus symbol indicates the commercial software in which the database was created and incorporated*,

± *BioTyper™—software from Bruker Daltonics, Bremen, Germany*.

¥ *-SARAMIS™ software (Spectral. ARchive And Microbial Identification System, originally developed by AnagnosTec, Potsdam-Golm, Germany and now acquired and redeveloped as Vitek-MS by bioMérieux, Marcy L'Etoile, France)*.

#### Parasites

In every reported case of parasite database approaches, Bruker BioTyper software tool and formic acid/acetonitrile extraction was applied. In the first such study, 56 clinical specimens belonging to 23 species of *Leishmania* were cultured, and promastigote pellets were utilized for database construction (Cassagne et al., 2014). Among 69 clinical isolates used for testing, only three samples were not identified. In a similar study, a database was constructed from four reference strains and the two clinical isolate were identified as *L. infantum* (Culha et al., 2014). In another study, using 19 enteric parasite *Blastocystis* isolates from 19 patients, a database for five subtypes was created and the remaining specimens were identified by matching (Martiny et al., 2014). Likewise, the differentiation of *E. histolytica* and *E. dispar* was demonstrated after establishment of the reference spectra and discriminating peaks were matched with the proteins identified through SDS-PAGE MALDI TOF MS based protein identification approach (Calderaro et al., 2015). Bruker BioTyper based database compilation were also reported for the food nematode *Trichinella* (Mayer-Scholl et al., 2016), *Trichomonas vaginalis* (Calderaro et al., 2016), *Acanthamoeba* spp. (Del Chierico et al., 2016) using a database for direct identification of trypanosomatids (Avila et al., 2016).

#### Toward “vector spectra reference database”

Kaufmann et al., were the first to report on utilization of SARAMIS premium software to create a reference database for two laboratory-reared *C. nubeculosus* biting midges (Kaufmann et al., 2011), and which was then extended to a larvae-specific database, screening of field collected samples, and applied for entomological surveys in Senegal, Africa. It was further reported as a means for discrimination of cryptic *Anopheles*, and demonstrated that the usefulness of mosquito eggs in species identification of field collected samples (Schaffner et al., 2014; Yssouf et al., 2014a). The Bruker Biotyper database has since been utilized to create a comprehensive “Vector specific reference spectra database” that includes spectra from leg proteins of 6 tick species, 30 mosquito species, one louse, one triatomine, one bed bug, and five flea species (Yssouf et al., 2013a,b, 2014b, 2015a). In addition, the package has been used to establish an aquatic developmental stage database starting from the larva stage to pupa of 6 mosquito species and detection of host blood meal and the presence of parasites (Niare et al., 2016; Laroche et al., 2017).

#### Pests

Despite the earlier proof of concept experiments for pests, the database approach was reported for only one plant pest in which Biotyper was utilized to distinguish evolutionary and morphologically close species of spider mites. Female adults of the Kanzawa (*Tetranychus kanzawai*), the two-spotted (*T. urticae*) spider mites and three other related species, namely *T. phaselus* Ehara (Tp), the bean red spider mite (*T. ludeni* Zacher) (Tl), and the tomato red spider mite (*T. evansi* Baker & Pritchard) (Te) were shown to be distinguishable. On the other hand, male adults and nymphs were reported to be non-distinguishable. Direct lysing of a single intact mite on target plates using double side carbon tape was also shown to generate spectra comparable to that of the extracts from 10 pooled individuals (Kajiwara et al., 2016). Recently, it was demonstrated that using MALDI MS, Tinkerbell LT, and its associated software MicroIDSys (ASTA Inc. Suwon, Korea), the larvae of the Korean apple pest, *Carposina sasakii*, could be effectively discriminated in about 15 min. Without such rapid identification methods, the export of these fruits might be hampered or rejected due to time-consuming pre-export inspection (Jeon et al., 2017).

## Dual identification of vectors, parasites, and meal source

MALDI MS profiling has also been shown to be an effective method for simultaneous identification of vectors and parasite species, as in case of simultaneous identification of *Borrelia crocidurae/Ornithodoros sonrai* and *Rickettsia* spp. in ticks (Fotso et al., 2014; Yssouf et al., 2015b). Direct spotting of haemolymph from a dissected tick leg allows for the simultaneous and direct species identification of ticks and associated pathogens, and useful in species identification of parasites and vector while leaving the vector remains available for other laboratory investigations (Fotso et al., 2014). MALDI MS profiling has further been successfully applied to determine the feeding patterns of mosquitoes up to 24 h post-blood meal (Niare et al., 2016). A future goal for the application of MALDI MS profiling in pest/parasites will be the rapid species identification of vectors, parasites and blood meal either through individual analysis or through simultaneous monitoring processes. The recent demonstration of rapid distinguishing of the *Plasmodium* infection status among *Anopheles stephensi* mosquitoes underscores that MALDI MS typing could be useful in entomological surveys including species-specific infection status (Laroche et al., 2017).

## Universal database and field samples

All the reported parasitology associated databases were developed as in-house databases and usually not accessable by other researchers, although several authors have agreed to supply the reference spectra upon request to other scientists possessing the same instrumentation and software tools. However, beyond a few initiations such as SpectraBank, there is no universal reference database such as protein database Swissprot/Uniprot for the purpose of species identification (Bohme et al., 2012). Recently, it was shown that the spectra generated from two different commercial MALDI MS instruments (Axima Confidence and Bruker Ultraflex III MS) could be analyzed in a single database (SARAMIS) in which a database was constructed for 20 species of phlebotomine sand flies based on measurements with Axima Confidence MS (Mathis et al., 2015). This underscores the possibilities of creation of online public reference databases that might be useful for any type of MALDI MS machines or spectral data formats. The online public database for parasitology is of great importance in terms of economy, time, and rapid analysis of samples collected from different geographical regions or hosts. The open source reference spectra database demands standardization of influencing parameters/processes affecting the sampling, such as developmental stage, specimen storage conditions, sample preparation/extraction methods, variations due to the spotting, instrumental, and post-measurement software settings.

## Conclusion

Despite the achievements of MALDI TOF MS in microbial species identification, the application in parasitology remains limited to *in-house* databases integrated into commercial software. There is no commercial software tool or reference database available for parasitology, however, most of the successful reports were based on the principles, procedures, software, and databases best described for microorganisms. The crucial factors influencing parasitology MALDI MS typing includes: specimen associated parameters (age, developmental stage, sex, differing body parts, fed state), sample preparation procedures (sample storage, protein extraction solvents, and methods, sample spotting method, and matrix), and measurement parameters. MALDI MS typing of field specimens will be helpful in creating a distribution map and evaluation of the spread of parasites associated with disease. Few reports describe the spectral differences among the geographically different populations (Dieme et al., 2014; Dvorak et al., 2014; Hoppenheit et al., 2014). However, one should remain cautious, as the reliability of MALDI TOF MS in phylogenetic analysis is yet to be proven. The establishment of open source software and databases might be useful in future parasitological surveys and for rapid assessment of real time infection status.

## Author contributions

JM conceived and wrote the manuscript. UR reviewed the manuscript.

### Conflict of interest statement

The authors declare that the research was conducted in the absence of any commercial or financial relationships that could be construed as a potential conflict of interest.
